# Associations between tobacco control mass media campaign expenditure and smoking prevalence and quitting in England: a time series analysis

**DOI:** 10.1136/tobaccocontrol-2017-053662

**Published:** 2017-06-30

**Authors:** Mirte A G Kuipers, Emma Beard, Robert West, Jamie Brown

**Affiliations:** 1 Department of Epidemiology and Public Health, University College London, London, UK; 2 Department of Public Health, Amsterdam Public Health Research Institute, Academic Medical Center, University of Amsterdam, Amsterdam, The Netherlands; 3 Research Department of Clinical, Educational and Health Psychology, University College London, London, UK

**Keywords:** smoking prevalence, mass media, campaigns, tobacco control, time-series, ARIMA

## Abstract

**Background:**

It has been established that mass media campaigns can increase smoking cessation rates, but there is little direct evidence estimating associations between government expenditure on tobacco control mass media campaigns and smoking cessation. This study assessed the association over 8 years between mass media expenditure in England and quit attempts, smoking cessation and smoking prevalence.

**Methods:**

Autoregressive integrated moving average modelling with exogenous variables (ARIMAX) was applied to monthly estimates from the Smoking Toolkit Study between June 2008 and February 2016. We assessed the association between the trends in mass media expenditure and (1) quit attempts in the last two months, (2) quit success among those who attempted to quit and (3) smoking prevalence. Analyses were adjusted for trends in weekly spending on tobacco by smokers, tobacco control policies and the use of established aids to cessation.

**Results:**

Monthly spending on mass media campaigns ranged from nothing to £2.4 million, with a mean of £465 054. An increase in mass media expenditure of 10% of the monthly average was associated with a 0.51% increase (of the average) in success rates of quit attempts (95% CI 0.10% to 0.91%, p=0.014). No clear association was detected between changes in mass media expenditure and changes in quit attempt prevalence (β=–0.03, 95% CI –2.05% to 2.00%, p=0.979) or smoking prevalence (β=–0.03, 95% CI –0.09% to 0.03%, p=0.299).

**Conclusion:**

Between 2008 and 2016, higher monthly expenditure on tobacco control mass media campaigns in England was associated with higher quit success rates.

## Introduction

The WHO’s Framework Convention on Tobacco Control mandates countries to promote ‘public awareness about the health risks of tobacco consumption and exposure to tobacco smoke, and about the benefits of the cessation of tobacco use and tobacco-free lifestyles’.^1^ Tobacco control mass media campaigns aim to raise the salience of the harmful effects of smoking, promote quitting, and often provide information on how to obtain help to quit.^2^ Tobacco control mass media campaigns are considered to be an important part of a comprehensive tobacco control strategy^3^ due to their potential impact^4–6^ by decreasing cigarette consumption^7 8^ and smoking prevalence^7–9^ and increasing quitting-related behaviours.^10–13^

In spite of the available evidence, there remains the crucial question as to what effect can be achieved for a given level of expenditure on tobacco control mass media campaigns by national governments.^14^ A review of 10 studies reported that mass media campaigns are cost-effective in terms of life years or quality-adjusted life-years gained.^15^ However, evidence is largely limited to studies of specific campaigns and expenditure over a relatively short period of time.^16–19^ Evaluations of expenditure over a more extended period have been conducted in the USA, where they found a positive association with calls to quitlines^20^ and a negative association with cigarette sales.^21^ Moreover, a freeze on mass media expenditure in the UK was associated with a decrease in the use of smoking cessation literature, quitline calls and hits on the national smoking cessation website.^10^ However, there is little evidence linking expenditure and actual quitting. Evaluations of specific campaigns such as No Smoking Day^22^ or Stoptober^17^ may provide different results from an analysis of mass media spending extending over a period of a year or more.

The US Centers for Disease Control (CDC) Best Practices for Comprehensive Tobacco Control Programs recommend an investment in tobacco control mass media campaigns of $0.65 to $1.95 per person per year.^23^ England has for the past 15 years had a strong tobacco control climate^24^ and has run mass media campaigns for most of that time, although not at the level recommended by the CDC. In recent years, spending on campaigns in England has varied markedly, including a moratorium on spending on major ‘above the line’ campaigns (that is, campaigns using traditional forms of media) when the Conservative-Liberal Democrat coalition came to power in 2010–2011.^10^ This ‘natural experiment’ provides a unique opportunity to study the impact of mass media spending across a wide range of values over a period of years.

This study aimed to assess the associations between changes in mass media tobacco control expenditure in England and changes in population-level quit attempt rates, smoking cessation rates and smoking prevalence. These outcome measures address the hypotheses that mass media campaigns may encourage smokers to attempt quitting, could lead to more successful smoking cessation and lower smoking prevalence. We included data on mass media expenditure before and after the 2010–2011 moratorium, which provided us with a large variation in expenditure data. With the use of the Smoking Toolkit Study, involving monthly cross sectional surveys of representative samples of the adult population of England, we were able to compare expenditure trends with trends in smoking behaviour while adjusting for important developments in tobacco spending, use of cessation aids and tobacco control over time. This provides information that complements previous evaluations of tobacco control mass media campaigns.^15^

## Methods

### Data and study population

Data were obtained from 165 420 individuals between June 2008 and February 2016 taking part in Smoking Toolkit Study (STS), a population survey of adults aged 16+ (www.smokinginengland.info). In the STS, each month a new sample of approximately 1800 adults aged ≥16 years is selected using a form of random location sampling. Individuals complete a face-to-face computer-assisted household interview survey with a trained interviewer. The STS samples have been shown to be nationally representative in their sociodemographic composition and proportion of smokers in the population 16+. Ethical approval was granted by the University College London ethics committee. Full details of the STS methods have been described elsewhere.^25^

### Measures

#### Smoking behaviour

Smoking was measured with the question: ‘Which of the following best applies to you?’, with response options: (1) ‘I smoke cigarettes (including hand-rolled) every day’, (2) ‘I smoke cigarettes (including hand rolled), but not every day’, (3) ‘I do not smoke cigarettes at all, but I do smoke tobacco of some other kind’, (4) ‘I have stopped smoking completely in the last year’, (5) ‘I have stopped smoking completely more than a year ago’, (6) ‘I have never been a smoker (ie, I have never smoked for a year or more)’. Last-year smokers were defined as individuals selecting answers 1 through 4. Individuals selecting options 1 through 3 were considered to be current smokers. Smoking prevalence was measured as the proportion of current smokers in the total population.

Quit attempts in the last 2 months were measured among last-year smokers who answered: ‘How many serious attempts to stop smoking have you made in the last 12 months?’, and if one or more attempts were reported: ‘How long ago did your most recent serious quit attempt start?’. We distinguished those who attempted to quit up to 2 months ago versus those who made no quit attempt or attempted to quit more than 2 months before the interview but were not successful. Last-year smokers who had successfully quit smoking more than 2 months before the interview were excluded for the analysis on quit success.

Successful quit attempts in last 2 months were defined as last-year smokers who attempted to quit in the last 2 months (defined as described above) who were still not smoking at the time of the interview. This only represents short-term success, but to go back further would have increased the risk of bias from forgotten quit attempts,^26^ and long-term quit success in large samples can be reliably estimated from short-term success. The relapse rate between 1 month and 1 year is approximately 70%, and between 1 year and 10 years is approximately 30%.^27^

#### Mass media expenditure

Data on tobacco control campaign expenditure in England were provided by Public Health England. Total spending on campaigns was provided for each period in which the campaign ran, which in many cases included multiple months. Spending (in million £) on ‘Smokefree’ campaigns, Stoptober campaigns and Health Harms campaigns were summed and monthly expenditure was extracted. Monthly totals included expenditure on TV, radio, print, cinema and online advertisements. In the months in which there was no campaign running and thus no campaign expenditure reported, Public Health England (PHE) confirmed that campaign expenditure could be regarded as effectively zero, and thus expenditure values were set to a nominal value of 0.01 (£10 000; zero values cannot be modelled). Expenditure figures diverge from those reported earlier (eg, in Action on Smoking and Health (ASH) publications^28^), as ‘lead generation’ expenditure was included in the ASH figures but not in the present study. Lead generation involved using databases to attempt to route individual smokers to stop smoking services.

#### Potential confounding variables

We included a variable reflecting the course of tobacco control policies in England. The value of the tobacco control variable increased by one unit with the introduction of each new policy in the study period (June 2008–February 2016) and was assigned to the month of when the policy was implemented. Over time, the variable values ranged from 1 to 7, with one point added when each of the following six tobacco control policies were implemented: (1) October 2009: pictorial warnings on cigarette packs, (2) October 2010: pictorial warnings on all tobacco products, (3) October 2011: ban on sale of tobacco from vending machines, (4) October 2013: ban advertising at the point of sale, (5) April 2013: ban on displaying cigarette packs in large shops, (6) April 2015: ban on displaying cigarette packs in small shops. As this variable was ‘differenced’ in the analysis (see Statistical Analysis), it amounted to an impulse of 1 in a month in which a new policy was introduced versus 0 in all other months.

Current smokers estimated their weekly spending on tobacco (in £). Weekly spending was adjusted for inflation using UK Consumer Price Index (CPI) information from the Office of National Statistics: adjusted spending = (spending/CPI)×100. Weekly spending was missing for survey waves between July 2009 and July 2010, for which a linear trend in mean spending is imputed.

The use of any cessation aids in a quit attempt including prescription medication (varenicline or bupropion), prescription nicotine replacement therapy (NRT) or electronic cigarettes (e-cigarettes) were measured among last-year smokers attempting to quit. The use of e-cigarettes, prescribed NRT, varenicline and bupropion have previously been shown to support successful quitting in this population.^29 30^ Use of NRT bought over the counter in quit attempts has not been found to increase quit rates in this population^29^ and was therefore not included.

### Statistical analysis

The analysis plan was registered on the Open Science Framework prior to data analysis (https://osf.io/gudrv/). An amendment was made to the analysis plan subsequently. The decision was made not to conduct the subgroup analyses due to the complexity of the overall results in terms of the number of lags and presence of autocorrelation.

Data were aggregated monthly as weighted means or proportions and analysed between April and July 2016 in R V.3.3.2. Means and proportions were weighted for gender, working status, prevalence of children in the household, age, social grade and region, as described in Fidler *et al*.^25^

We applied Autoregressive Integrated Moving Average (ARIMA) with Exogenous Input (ARIMAX) modelling, which is comparable to methods used in other tobacco control evaluation studies.^9 12^ ARIMAX is similar to ARIMA analysis, in that it uses forecasts based on prior values in the time series (autoregressive terms (AR)) and errors made by previous predictions (moving average terms (MA)). Additionally, ARIMAX can assess associations between multiple time series, allowing control for other processes taking place over time. We followed a standard ARIMAX modelling approach which is given in more detail in the preregistered analysis plan.^31^

Time series analysis requires stationary data. To achieve this, the series are first log transformed to stabilise the variance and, if required, are differenced and seasonally differenced. The differenced value of Y at month t would be Y_t–_Y_t-1_ and the seasonally differenced value would be Y_t–_Y_t-12_. Differencing attempts to remove underlying linear and cyclical trends. The autocorrelation and partial autocorrelation functions were examined to determine the seasonal and non-seasonal MA and AR. To identify the most appropriate transfer function for the continuous explanatory variables, the sample cross-correlation function was checked for each ARIMAX model. ARIMAX models with several combinations of values for p (the number of autoregressive terms) and q (the number of lagged forecast errors), and several lags were run, and the models with the best fit were presented. Additionally, a model without lag for mass media expenditure was estimated. A detailed explanation of the model selection is available in the online supplementary material. Coefficients can be interpreted as estimates of the percentage change in the mean outcome of interest across the study period for every percentage increase in mass media expenditure above the monthly mean across the study period. In the tables, the coefficients were multiplied by 10 to represent the change with a 10% increase in expenditure. STROBE guidelines were followed.^32^

10.1136/tobaccocontrol-2017-053662.supp1 Supplementary material 1

In sensitivity analyses, we tested models without lags for all covariates and models with additional AR and MA terms. In all sensitivity analyses, the results for mass media expenditure were compared with the results of the main analysis.

## Results

**Figure 1:**
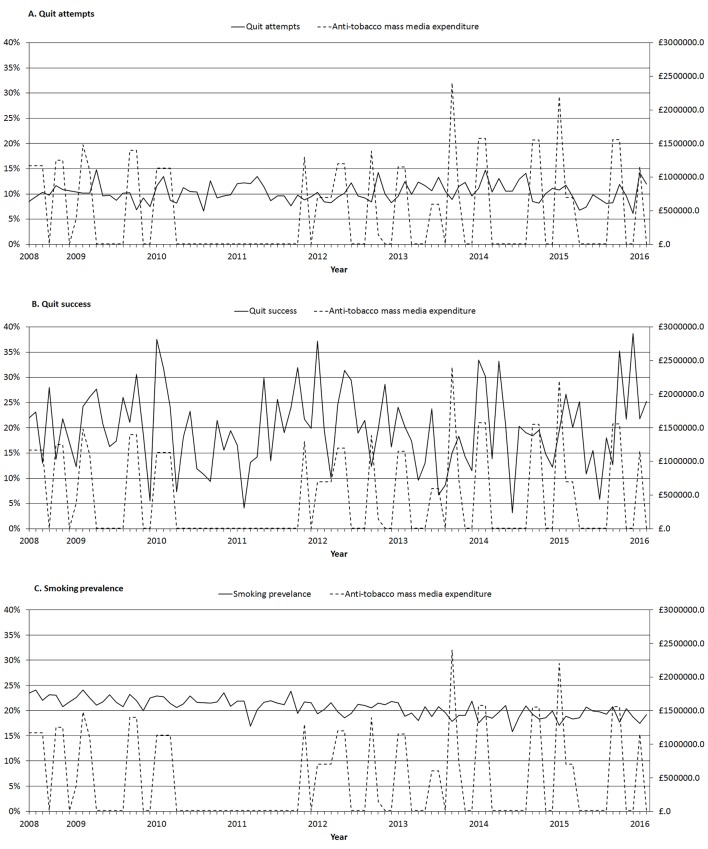
Weighted monthly trends of (A) quit attempts in the last 2 months, (B) quit success in those who attempted in the last 2 months and (C) smoking prevalence in the general population and the expenditure on mass media tobacco control campaigns in pound per month.

**Figure 2:**
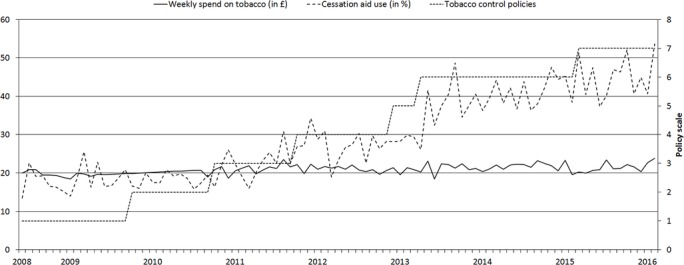
Weighted monthly trends in the use of e-cigarettes, prescription nicotine replacement therapy and prescription medication by smokers who attempted to quit in the last year (in %), weekly spend on tobacco by smokers (in £) and tobacco control policies.

Descriptive statistics are presented in table 1, figure 1 and figure 2. Over the period June 2008 to February 2016, Public Health England spent £43.2 million on tobacco control mass media campaigns. As depicted in figure 1, expenditure varied over the years, and was generally high in 2008 and 2009, after which it dropped to zero during a moratorium on spending. Expenditure resumed in 2012 to 2015. Table 1 and figure 1 show that the weighted smoking prevalence over the total study period was 20.6% (95% CI 20.4% to 20.8%) and that it decreased over time. The 2-month quit attempt rate (percentage of smokers having made a quit attempts in the past 2 months) over the total period was 10.3% (95% CI 9.9% to 10.6%) and somewhat increased over time, while quit success among those who attempted to quit did not show a clear linear trend and fluctuated around 20% (table 1 and figure 1). The use of cessation aids rapidly increased (table 1 and figure 2).

**Table 1:** Description of national tobacco control mass media expenditure and weighted individual level variables by survey period, in % with 95% CI, unless otherwise specified

	Total June 2008–February 2016	June 2008–December 2010	January 2011–December 2013	January 2014–February 2016
Mass media expenditure (total in million £)	43.2	15.2	14.0	14.1
In the total population, N	1 65 420	56 355	65 443	43 622
Current smoking	20.6 (20.4–20.8)	22.1 (21.8–22.5)	20.4 (20.1–20.7)	19.0 (18.6–19.4)
In smokers, N	37 013	13 120	14 331	8487
Quit attempts	10.3 (9.9–10.6)	10.0 (9.5–10.6)	10.4 (9.9–11.0)	10.4 (9.7–11.1)
2-month quit success in those who attempted to quit	19.9 (18.5–21.3)	20.2 (18.0–22.7)	18.8 (16.7–21.1)	21.0 (18.2–24.1)
Cessation aids use	28.2 (27.4–29.1)	18.7 (17.6–19.9)	28.8 (27.5–30.2)	42.6 (40.6–44.5)
Weekly spend tobacco (mean in £, 95% CI)	20.9 (20.7 to 21.1)	19.9 (19.5 to 20.2)	21.1 (20.8 to 21.4)	21.6 (21.2 to 22.0)

Table 2 shows the percentage change in quit attempts for a 10% increase in mass media expenditure above the monthly average, as estimated in the ARIMAX models. Mass media expenditure was not significantly associated with concurrent change in quit attempts rates (per cent change −0.04, 95% CI −0.63 to 0.54, p=0.883), nor was it significantly associated with quit attempts 2 months later (per cent change −0.05, 95% CI −0.67 to 0.56, p=0.861). Results were similar in the unadjusted and adjusted models. Table 2 also shows that the quit attempt rate was not associated with smokers’ weekly spend on tobacco or with tobacco control policies.

**Table 2:** Estimated percentage change in quit attempts (the proportion of smokers who attempted to quit in the past 2 months) per 10% change in mass media expenditure from ARIMAX models

Quit attempts	Unadjusted	Adjusted for covariates in table
	Percentage change per 10% change in the exposure (95% CI), p Value	Percentage change per 10% change in the exposure (95% CI), p Value
Model 1		
Mass media expenditure (lag 0)	−0.04 (-0.63 to 0.54), 0.883	−0.03 (-0.62 to 0.56), 0.931
Weekly spend tobacco (lag 4)		−0.51 (-2.89 to 1.87), 0.677
Tobacco control policies		0.06 (-0.49;0.62), 0.830
Best fitting model	ARIMAX (0, 1, 1) (0, 0, 0)	ARIMAX (0, 1, 1) (0, 0, 0)
Non-seasonal (p)—AR	NA	NA
—MA	<0.001	<0.001
Seasonal (p)—AR	NA	NA
—MA	NA	NA
R^2^	0.010	0.012
Model 2		
Mass media expenditure (lag 2)	−0.05 (-0.67 to 0.56), 0.861	−0.03 (-2.05 to 2.00), 0.979
Weekly spend tobacco (lag 4)		−0.51 (-2.94 to 1.93), 0.684
Tobacco control policies		−0.06 (-0.50 to 0.62), 0.831
Best fitting model	ARIMAX (0, 1, 1) (0, 0, 0)	ARIMAX (0, 1, 1) (0, 0, 0)
Non-seasonal (p)—AR	NA	NA
—MA	<0.001	<0.001
Seasonal (p)—AR	NA	NA
—MA	NA	NA
R^2^	0.010	0.012

The assumption of normally distributed errors was met. When the lag for weekly tobacco spend was set to zero, results for mass media were similar in model 1 (β=−0.04 (–0.63 to 0.54), p=0.882) or in model 2 (β=−0.05 (-0.66; to 0.56), p=0.864). Addition of MA or AR terms did not improve the models.

AR, autoregressive terms; ARIMAX, Autoregressive integrated moving average modelling with exogenous variables;MA, moving average terms.

Table 3 shows ARIMAX results for quit success. With every 10% increase in mass media expenditure above the monthly average, successful smoking cessation among quitters immediately increased by 0.55% (95% CI 0.15 to 0.96, p=0.007) in the unadjusted model and by 0.51% (95%CI 0.10 to 0.91, p=0.014) in the adjusted model. A lag of 1 month for mass media expenditure, although with a lower model fit, led to a comparable increase in quit success (per cent change 0.49, 95% CI 0.10 to 0.87, p=0.013). Covariates did not show significant associations with quit success.

**Table 3:** Estimated percentage change in quit success (the proportion successful quitters among those who made an attempt in the past 2 months) per 10% change in mass media expenditure from ARIMAX models

Quit success	Unadjusted	Adjusted for covariates in table
	Percentage change per 10% change in the exposure (95% CI), p Value	Percentage change per 10% change in the exposure (95% CI), p Value
Smoking cessation		
Mass media expenditure (lag 0)	0.55 (0.15 to 0.96), 0.007	0.51 (0.10 to 0.91), 0.014
Weekly spend tobacco (lag 0)		−16.83 (-37.41 to 3.75), 0.109
Cessation aid use (lag 4)		2.11 (-1.51 to 5.73), 0.254
Tobacco control policies		−0.15 (-2.09 to 1.79), 0.878
Best fitting model	ARIMAX (0, 1, 1) (0, 0, 0)	ARIMAX (0, 1, 1) (0, 0, 0)
Non-seasonal (p)—AR	NA	<0.001
—MA	<0.001	NA
Seasonal (p)—AR	NA	NA
—MA	NA	NA
R^2^	0.075	0.112

Additional MA (0, 1, 2) or AR (1, 1, 1) terms were not significant. The assumption of normally distributed errors was met. When all lags were set to zero in the adjusted model, similar results were found for mass media (β=0.50 (0.10 to 0.90), p=0.015). A lag of 1 month for mass media expenditure, although with a considerably worse fit, led to a comparable increase in quit success (β=0.49 (0.10 to 0.87), p=0.013).

AR, autoregressive terms; ARIMAX, Autoregressive integrated moving average modelling with exogenous variables;MA, moving average terms.

Table 4 shows ARIMAX results for smoking prevalence. A significant decrease in smoking prevalence in response to mass media expenditure was not observed and results were the same for models without a lag and with a lag of 2 months (percent change −0.03, 95% CI −0.09 to 0.03, p=0.299). Covariates did not show significant associations with smoking prevalence, although the use of cessation aids tended to be associated with lower smoking rates (percent change −0.18, 95% CI −0.39 to 0.03, p=0.097).

**Table 4:** Estimated percentage change in smoking prevalence (the proportion of smoking in the general population) per 10% change in the exposure from ARIMAX models

Smoking prevalence	Unadjusted	Adjusted for covariates in table
	Percentage change per 10% change in the exposure (95% CI), p Value	Percentage change per 10% change in the exposure (95% CI), p Value
Model 1		
Mass media expenditure (lag 0)	−0.03 (-0.09 to0.03), 0.275	−0.03 (-0.09 to 0.02), 0.258
Weekly spend tobacco (lag 1)		0.08 (-0.09 to 0.25), 0.371
Cessation aid use (lag 1)		−0.18 (-0.38 to 0.03), 0.096
Tobacco control policies		−0.54 (-1.44 to 0.36), 0.238
Best fitting model	ARIMAX (0, 1, 1) (0, 0, 0)	ARIMAX (0, 1, 1) (0, 0, 0)
Non-seasonal (p)—AR	NA	NA
—MA	<0.001	<0.001
Seasonal (p)— AR	NA	NA
—MA	NA	NA
R^2^	0.465	0.527
Model 2		
Mass media expenditure (lag 3)	−0.03 (-0.09 to 0.03), 0.269	−0.03 (-0.09 to 0.03), 0.299
Weekly spend tobacco (lag 1)		−0.08 (-0.10 to 0.25), 0.379
Cessation aid use (lag 1)		−0.18 (-0.39 to 0.03), 0.097
Tobacco control policies		−0.54 (-1.44 to 0.35), 0.235
Best fitting model	ARIMAX (0, 1, 1) (0, 0, 0)	ARIMAX (0, 1, 1) (0, 0, 0)
Non-seasonal (p)—AR	NA	NA
—MA	<0.001	<0.001
Seasonal (p)—AR	NA	NA
—MA	NA	NA
R^2^	0.465	0.527

The assumption of normally distributed errors was met. When lags of tobacco spending and cessation use were set to zero very similar results were found for mass media in model 1 (β=−0.03 (-0.09 to 0.02), p=0.238) and in model 2 (β=−0.03 (-0.10 to 0.03), p=0.278). Additional MA (0, 1, 2) or AR (1, 1, 1) terms were not significant.

AR, autoregressive terms; ARIMAX, Autoregressive integrated moving average modelling with exogenous variables;MA, moving average terms.

## Discussion

### Key findings

Government spending on mass media campaigns ranged from nothing to £2 400 000 per month. No clear association was detected between changes in mass media expenditure and changes in concurrent quit attempts, quit attempts in the following 2 months or smoking prevalence. However, every 10% increase in mass media expenditure above the monthly average was associated with a 0.51% increase from the average in short-term success of quit attempts.

### Study limitations

This study had a number of limitations. First, the association observed between expenditure and quit success may not be causal, but due to unmeasured confounding. We attempted to control for major factors that could affect success of quit attempts at a population level, including the rise in popularity of e-cigarettes.^33^ However, it remains possible that our measures were not sufficiently precise or that there was residual confounding. For example, campaigns may influence social norms to be less accepting of smoking,^34^ which increases support for more tobacco control investment and may in turn increase mass media expenditure. However, Granger causality tests were performed as a part of the ARIMAX analyses and found weak exogeneity between mass media expenditure and all three smoking outcomes.

Second, we relied on self-reports of smoking and quitting. These may be subject to error and bias. For example, failed quit attempts tend to be forgotten relatively quickly, and this could weaken our ability to detect an effect on quit attempts. On the other hand, these measures have shown reliable associations with other factors that influence quitting, such as use of e-cigarettes^33^ and cigarette warning labels.^35^

Third, while the sample was intended to be representative and had characteristics that were similar to other large population samples in England,^25^ it is likely that there is bias in the type of people who respond to these kinds of surveys. This is an important area for study as it appears that there is a reduced willingness on the part of the general public to take part in surveys.^36^ The results of the current study may be biased if participants are more engaged in societal issues and perhaps more responsive to mass media campaigns than non-participants.

Fourth, the findings represent trends in England over a particular time period with particular kinds of mass media campaigns, and an optimal level of expenditure could not be identified. It is possible that different results would be obtained in other circumstances with different campaign strategies.^7 11 37^ This means that the effectiveness of England’s future mass media expenditure and expenditure by foreign governments is not guaranteed by our results. However, England’s mass media campaign strategy is broadly similar—including use of similar material—to other countries, such as Australia. The fact that the association with quit success was found over a relatively long period of study with a variety of different types of campaigns, probably gives a broad indication of what can be achieved by a well-informed campaign strategy. In 2004–2010, 89% of tobacco control mass media campaigns was for smoking cessation, among which half contained how-to-quit messages.^2^

### Interpretation

Mass media expenditure in England over the period of the study was associated with more successful quitting among those who attempted to stop. This is in line with findings from a controlled trial in four regions in central and northern England, which found that an antismoking TV campaign that provided tips on how to prevent relapse was effective in helping prevent relapse.^13^ Relapse prevention tips were also found to be helpful in a study among ex-smokers in the USA.^38^ Another US study found that tips on how to quit from former smokers increased quit success among smokers making a quit attempt.^39^ Quit success may be increased due to mass media campaigns sustaining motivation to persist with the quit attempt by maintaining the salience of quitting or making quitting appear more normative.^4 34^ Moreover, smokers may more often seek professional help as a result of how-to-quit messages, such as behavioural or cognitive therapy, support groups, quitlines or other forms of professional support.^12^ This was demonstrated by Langley *et al*,^10^ showing that stopping mass media spending significantly reduced the number of literature requests, quitline calls and website hits.

If the relationship between mass media expenditure and quit success found in this study is causal, we can estimate that every 10% increase in expenditure would result in a 0.51% increase in short-term success rates, other things being equal. The mean quit success rate over the studied period would have been 19.98% instead of 19.87% if mass media expenditure was 10% higher. Over the study period, the monthly average expenditure on mass media campaigns was £465 054, or £5 580 644 per year, and according to the STS data 35.3% of smokers made a quit attempt in the last year. Our data showed that 19.9% of these quit attempts were successful after 2 months, and according to previous studies 55% of these quit attempts remained successful after 6 months^27^. With 8 million smokers in England, this amounts to 308 638 people (8 000 000×0.353×0.199×0.55). Thus, our findings suggest that if spending on mass media campaigns were increased by £1 million per year, there would have been an additional 5129 short-term ex-smokers. Applying an adjustment for subsequent relapse this would translate to 2500 people who stopped smoking permanently.^27^ Assuming that most smokers who would have stopped were in middle age, this amounts to 2903 additional life years gained at a cost of £344 (95% CI 193 to 1757) per life year using a standard annual discount rate of 3.5%.^27^ This incremental cost-effectiveness ratio of £344 is comparable with findings from previous cost-effectiveness studies on mass media campaigns.^16 17 40–42^

The failure to find a clear association of expenditure with quit attempts seems to conflict with previous studies.^4^ In fact, we cannot conclude that there was no effect of mass media campaign expenditure on quit attempts due to a wide confidence interval (ranging from −2.05 to 2.00), and previous studies have yielded mixed results.^4 17 39 43–45^ The circumstances in which mass media campaigns may increase quit attempts or not requires further study.

We did not find a significant association between mass media expenditure and smoking prevalence, although the association was in the expected direction. If the association we found with quit success is causal, the impact on smoking prevalence would only result in a 0.03% reduction in prevalence which we would be unlikely to detect with our sample size.

## Implications

Mass media expenditure is cost-effective at much lower costs than generally accepted thresholds for public health interventions: for example £20 000 as stated in the UK’s National Institute for Health and Care Excellence guidelines.^46^ Mass media expenditure in England has not increased since lifting the 2011 moratorium, and the English Department of Health announced a budget of £4 million for 2016/2017, compared with £5.3 million in 2015/2016, a cut of 25%.^47^ This could lead to 3251 fewer long-term ex-smokers. Increased expenditure on mass media campaigns, on the other hand, has the potential to reduce smoking, to gain life years and to have a positive return on investment.^48^

## Conclusions

Between 2008 and 2016, higher expenditure on tobacco control mass media campaigns in England was associated with an increase in quit success rates. An association with quit attempts and smoking prevalence was not clearly established. For an additional million pounds spent per year over and above expenditure across the period, we estimate there could have been 2500 additional permanent ex-smokers.

**What this paper adds**

- While it has been established that mass media campaigns can increase smoking cessation rates, there is little direct evidence estimating associations between government expenditure on tobacco control mass media campaigns and smoking cessation. The current study provides policy-makers with estimates of the impact of governmental investment in tobacco control mass media campaigns.
- Although no clear association was detected between changes in mass media expenditure and changes in quit attempt prevalence or smoking prevalence, higher expenditure on mass media campaigns as a component of a government tobacco control strategy was associated with higher quit success rates.
