# Supplementary material for: Associations between tobacco control mass media campaign expenditure and smoking prevalence and quitting in England: a time series analysis
Source: Tob Control. 2017 Jun 30;27(4):455–62. doi: 10.1136/tobaccocontrol-2017-053662 (PMC6047146; doi:10.1136/tobaccocontrol-2017-053662)
Supplement: Supplementary material 1 [file tobaccocontrol-2017-053662supp001.pdf]

Associations between anti-tobacco mass-media campaign expenditure and smoking prevalence and quitting in England: a time series analysis

Mirte AG Kuipers, Emma Beard, Robert West, Jamie Brown

ONLINE SUPPLEMENT

ARIMAX procedure and model selection

The analysis proceeded by first assessing each time series for outlying values which may have biased the results. No outliers were found on any of the included variables. We assessed the presence of exogeneity using the Granger Causality test. Granger causality tests were not violated. Plots of the differenced data and unit root tests (i.e. Osborn-Chui-Smith-Birchenhall test and Kwiatkowski, Phillips, Schmidt, and Shin (KPSS) test) were used to determine the number of differences required for the time series to be made stationary (1, 2). Time-series showed some non-stationarity and were differenced once (i.e. the value of the series at each point in time was replaced by the value of the difference between that month and the previous month) and log-transformed (3). No significant autocorrelation at seasonal lags was found, and time-series were therefore not seasonally differenced (i.e. the change between a month in one year and the next).

Due to differencing, the policy variable took the values 0 or 1, reflecting the points at which new policies were introduced. Due to the dichotomy of the variable it was included in the model as a binary explanatory variable. Mass-media expenditure, spending on tobacco and cessation aid use were included as continuous explanatory variables. To identify the most appropriate transfer function for the continuous explanatory variables the sample cross-correlation function (CCF) was checked for each ARIMAX model, with pre-whitened data (4). Pre-whitening removes autocorrelation in the input series that may causes spurious cross-correlation effects and therefore aids interpretation. For quit attempts none of the explanatory variables seemed to have lagged effects according to the CCF. For mass-media expenditure higher order lags were found for both quit success and smoking prevalence, which indicated that there may be some lag in the association, but may also reflect noise.

Additional checks were run by comparing univariate ARIMAX models with variations for the transfer function using the Akaike Information Criterion (AIC). For quit attempts, a transfer function of (2,0) (lag of two months) for mass-media expenditure was found to fit best and (4,0) for spending on tobacco. For quit success, transfer functions of (0,0) were found for mass-media expenditure and spending on tobacco, and (4,0) for cessation aids use. For smoking prevalence (3,0) was found for mass-media expenditure. According to AIC a transfer function of (5,0) had the best fit for tobacco spending and cessation aids. However, as the models for (2,0), (3,0) and (4,0) were much worse than (1,0), a transfer function of (5,0) is not likely to reflect a better fit but rather noise in the data. A parsimonious model with transfer functions of (1,0) for both variables was therefore used instead.

To determine the initial values of the AR and MA terms for the baseline models, the autocorrelation function (ACF) and partial autocorrelation function (PACF) were assessed. ACF and PACF plots suggested one MA term and no AR terms for all three outcomes (0,1,1). Additional models with various fitted AR and MA terms were then compared to this baseline model using the AIC. According to the Box-Jenkins method, in ARIMA (p, d, q) the value of

p and q should be 2 or less or the total number of parameters should be less than 3 (5). Therefore, we only checked ARIMAX models for p and q values of 3 or less. Despite the ACF/PACF plot information, in three models (0,1,1) was not found as the best fitting model according to AIC, but a (0,1,2) model instead. This was the case for the unadjusted model for quit attempts, and both the adjusted and unadjusted models for smoking prevalence. However, in all cases the second MA term was not significant and did not lead to different results for the association with mass-media expenditure. To keep the models parsimonious a (0,1,1) model was used.

The ACF for the residuals of the best fitting models was checked for additional correlation (thus the need for additional MA/AR seasonal or non-seasonal terms) and the coefficients of the correlation terms assessed for significance and whether they fell within the bounds of stationarity and invertibility (3, 6). Minor residual autocorrelation was found for quit attempts and smoking prevalence, but additional MA/AR seasonal or non-seasonal terms did not improve the model.

The Ljung-Box test for white noise was also used to statistically evaluate the degree to which the residuals were free of serial correlation (7), and in the final models residuals were assessed for normality. All Ljung-Box test p-values were non-significant, although for quit attempts and quit success borderline significant p-values were found. However, as stated above, additional MA/AR seasonal or non-seasonal terms did not improve the model.

Coefficients are reported along with their 95% confidence intervals (95% CIs), and a pseudo-R-squared calculated as the squared correlation of fitted to actual values. STROBE guidelines were followed throughout (8).

References

1. Lee D, Schmidt P. On the power of the KPSS test of stationarity against fractionally-integrated alternatives. Journal of Econometrics. 1996;73(1):285-302.
2. Osborn DR, Chui AP, Smith JP, Birchenhall CR. Seasonality and the order of integration for consumption. Oxford Bulletin of Economics and Statistics. 1988;50(4):361-77.
3. Yaffee R. An Introduction to Forecasting Time Series with Stata. New York: Taylor & Francis; 2012.
4. Cryer JC, K-S. Time series analysis - with applications in R. London: SpringerVerlag New York; 2008.
5. Box GE, Jenkins GM, Reinsel GC, Ljung GM. Time series analysis: forecasting and control: John Wiley & Sons; 2015.
6. Yaffee RA, McGee M. An introduction to time series analysis and forecasting: with applications of SAS® and SPSS®: Academic Press; 2000.
7. Montgomery DC, Jennings CL, Kulahci M. Introduction to time series analysis and forecasting: John Wiley & Sons; 2015.
8. Von Elm E, Altman DG, Egger M, Pocock SJ, Gøtzsche PC, Vandenbroucke JP, et al. The Strengthening the Reporting of Observational Studies in Epidemiology (STROBE) statement: guidelines for reporting observational studies. Preventive Medicine. 2007;45(4):247-51.
